# Fragmentation of DNA affects the accuracy of the DNA quantitation by the commonly used methods

**DOI:** 10.1186/1480-9222-15-5

**Published:** 2013-02-13

**Authors:** Tatiana Sedlackova, Gabriela Repiska, Peter Celec, Tomas Szemes, Gabriel Minarik

**Affiliations:** 1 Institute of Molecular BioMedicine, Faculty of Medicine, Comenius University, Sasinkova 4, Bratislava, 811 08, Slovakia; 2 Department of Molecular Biology, Faculty of Natural Sciences, Comenius University, Mlynska dolina, Bratislava, 842 15, Slovakia; 3 Geneton Ltd, Cabanova 14, Bratislava, 841 02, Slovakia

**Keywords:** DNA fragmentation, DNA quantitation, Spectrophotometry, PicoGreen, qPCR

## Abstract

**Background:**

Specific applications and modern technologies, like non-invasive prenatal testing, non-invasive cancer diagnostic and next generation sequencing, are currently in the focus of researchers worldwide. These have common characteristics in use of highly fragmented DNA molecules for analysis. Hence, for the performance of molecular methods, DNA concentration is a crucial parameter; we compared the influence of different levels of DNA fragmentation on the accuracy of DNA concentration measurements.

**Results:**

In our comparison, the performance of the currently most commonly used methods for DNA concentration measurement (spectrophotometric, fluorometric and qPCR based) were tested on artificially fragmented DNA samples. In our comparison, unfragmented and three specifically fragmented DNA samples were used.

According to our results, the level of fragmentation did not influence the accuracy of spectrophotometric measurements of DNA concentration, while other methods, fluorometric as well as qPCR-based, were significantly influenced and a decrease in measured concentration was observed with more intensive DNA fragmentation.

**Conclusions:**

Our study has confirmed that the level of fragmentation of DNA has significant impact on accuracy of DNA concentration measurement with two of three mostly used methods (PicoGreen and qPCR). Only spectrophotometric measurement was not influenced by the level of fragmentation, but sensitivity of this method was lowest among the three tested. Therefore if it is possible the DNA quantification should be performed with use of equally fragmented control DNA.

## Background

Circulating nucleic acids are currently studied as a potential diagnostic marker for oncological diseases as well as in relation to non-invasive prenatal diagnosis. Substantial fragmentation and low concentrations are limiting characteristic features of circulating nucleic acids (cNA). According to a recent study, the cNA are present in the circulation at sizes lower than 1200 bp and most of cNA molecules are clustered into two peaks, first at approximately 162 bp and second at 340 bp, representing a dominant and a minor peak [1]. These molecules are released from apoptotic cells after the programmed enzymatic cleavage process during apoptosis [2]. On the other hand, the fragment lengths of circulating nucleic acids vary in size in cases of malignant disease, because they are released from apoptotic cells as well as necrotic cells [3,4]. The second mentioned limiting characteristic of cNA is its low concentration. The accuracy of DNA quantification is crucial for the success of following downstream applications such as (q)PCR, sequencing and cloning. Commonly used methods of DNA concentration measurements are the evaluation of the intensity of a band on an agarose gel, fluorescence measurements using various DNA-binding dyes and measurements of UV absorbance at 260 nm [5], with the latter being the most commonly used [6,7]. The disadvantages of the latter method are that the absorbance measurement at 260 nm includes signals of a double-stranded and single-stranded DNA oligonucleotides and free nucleotides, the fact that it does not distinguish between DNA and RNA, and that it has a low sensitivity, reaching 1 ng/μl [6,8,9]. In contrast, fluorescent dyes selectively measure only double-stranded DNA and are much more sensitive [8,9]. The most commonly used fluorescent dyes are Hoechst 33258 and PicoGreen. Hoechst 33258 allows the detection and quantitation of DNA at concentrations as low as 10 pg/μl [9,10]. The measurement of concentration using PicoGreen, which is currently very popular, allows the detection of dsDNA in a final concentration as low as 25 pg/μl [8,9]. The disadvantage is that the concentration assessment by fluorescent dyes underestimates the concentration of double-stranded DNA with a size less than 23 kbp [6]. Another method used for DNA quantification is qPCR [11,12]. This is a good choice for qualitative as well as quantitative analysis of DNA because of its high sensitivity and specificity for typical molecular applications. The use of multi-copy genes, such as rDNA genes and Alu repeats, as qPCR targets can improve the qPCR sensitivity above the limited sensitivity of ordinary PCR [13,14], as well as fluorometric methods up to 1 picogram of human DNA [15]. The aim of our study was to determine whether the degree of DNA fragmentation affects the measurement of DNA concentration with the three most commonly used methods - spectrophotometry, fluorometry and qPCR. Because of specific sizes of cNA fragments isolated from plasma samples, we decided to compare measurements of unfragmented samples (~25 kb fragments) with artificially fragmented DNA samples at three targeted fragment sizes - 1500 bp, 500 bp and 150 bp. These three sizes should cover the whole sample as well as predominant cNA fractions.

## Results

**Figure 1:**
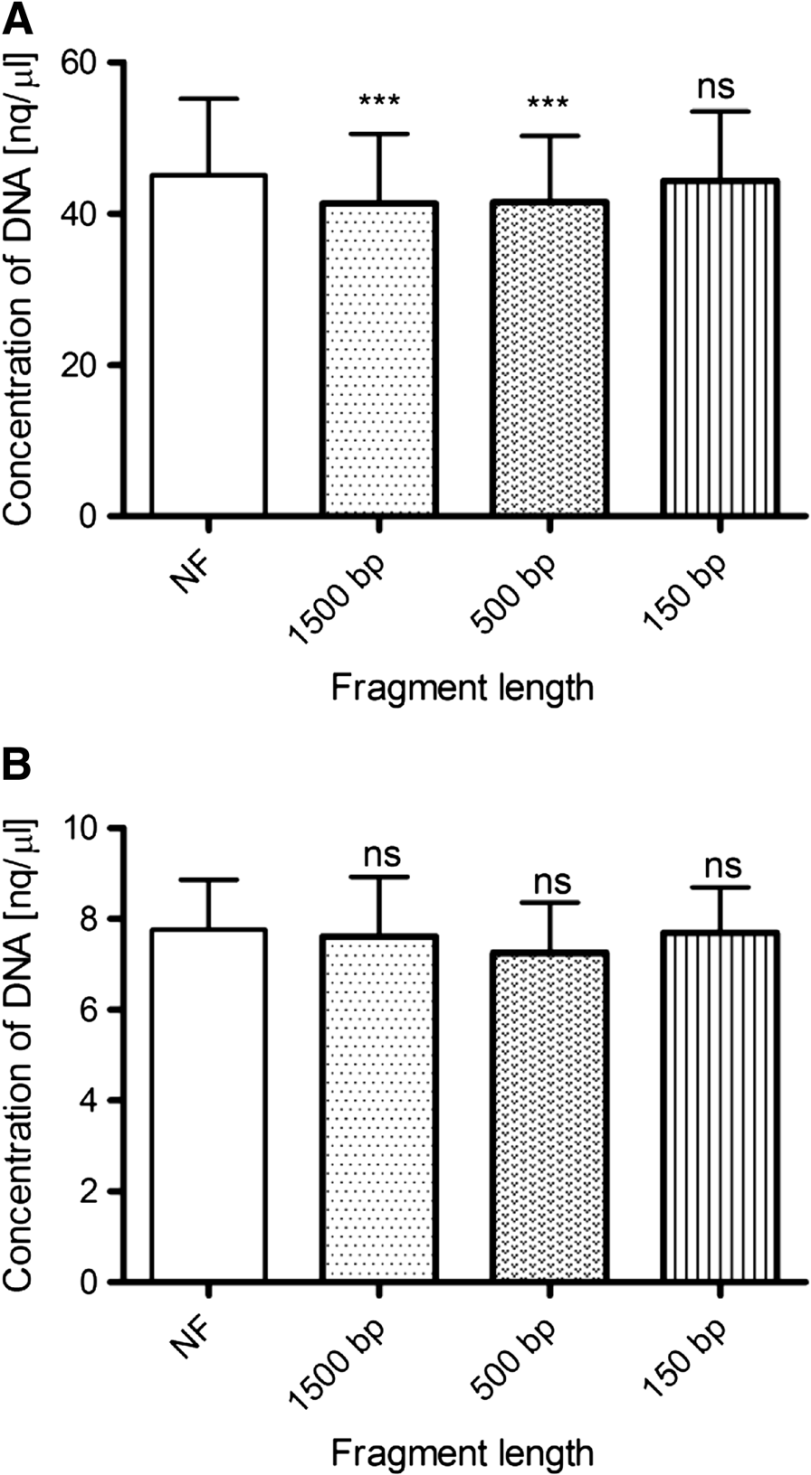
**UV spectrophotometric quantification of DNA by NanoDrop.** (**A**) Quantification of undiluted samples showed that the DNA quantity in the samples with the length of fragments approx. 1500 bp and 500 bp significantly decreased compared to concentration of unfragmented samples. There was no difference between the concentration of unfragmented DNA samples and specimens with the fragment length of 150 bp. (**B**) The DNA concentration of 10-fold diluted samples was not affected by the level of fragmentation. ns, statistically non-significant; ***, p< 0.0001.

DNA quantification by the spectrophotometric measurement of absorbance at 260 nm was performed in undiluted and 10-fold diluted samples. The 100-fold and 1000-fold diluted samples concentrations could not be measured due to concentrations below the detection limit of this method. Measurements of undiluted samples showed that the DNA quantities in samples with the length of fragments of approximately 1500 bp and 500 bp were slightly decreased compared to the concentration of unfragmented samples and those with fragments of approximately of 150 bp. This decrease in DNA concentration was statistically significant (p< 0.001). There was no difference between the concentration of unfragmented DNA samples and specimens with fragment peaks at 150 bp. DNA concentration was not affected by the level of fragmentation in the 10-fold diluted sample. Purity of DNA in all samples was determined based on the A_260/280_ ratio measured by spectrophotometry. The A_260/280_ ratio ranged from 1.83 ± 0.06 to 1.90 ± 0.04 for undiluted samples and from 1.93 ± 0.19 to 1.94 ± 0.23 for 10-fold diluted samples (Figure 1).

**Figure 2:**
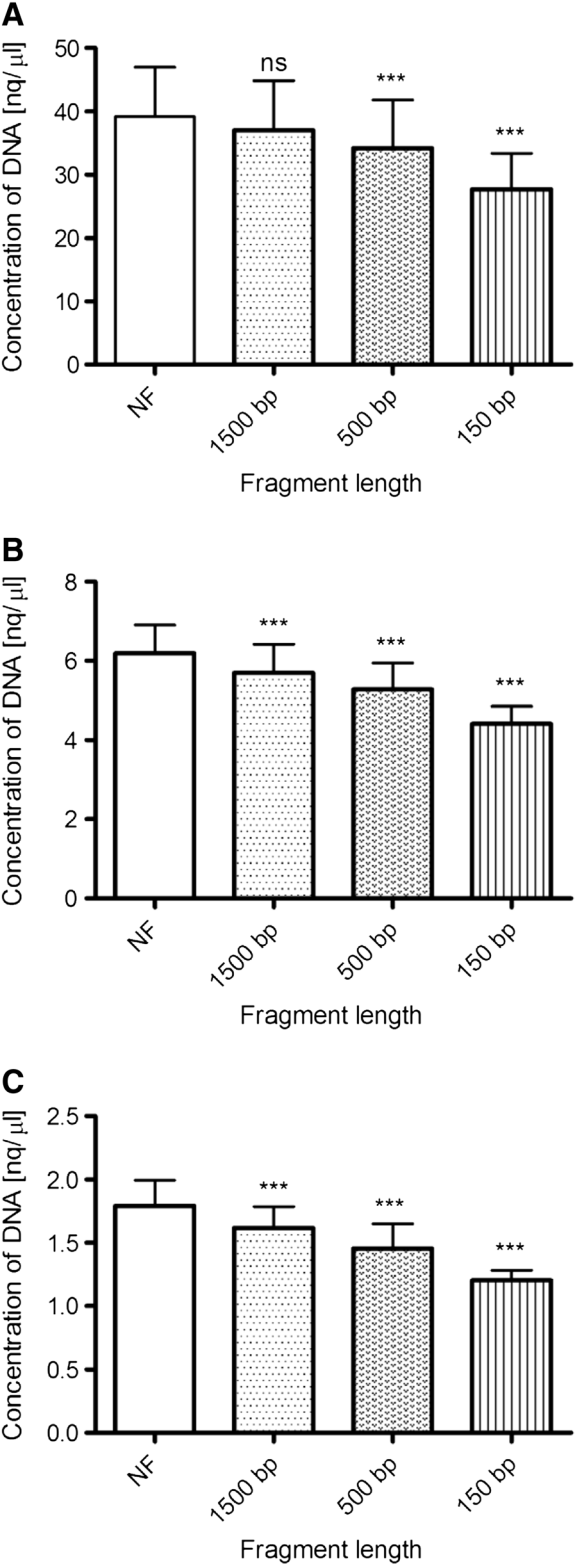
**Measurement of DNA concentration by PicoGreen.** (**A**) The DNA quantity of 10-fold diluted samples decrease as degree of fragmentation increase. (**B**) The concentration of DNA of 100-fold diluted samples is affected by level of the fragmentation in all different fragment lengths. (**C**) The DNA concentration of 1000-fold samples is also affected by degree of the fragmentation in all cases of fragment lengths. ns, statistically non-significant; ***, p< 0.0001.

Regarding the DNA quantification by PicoGreen fluorescent dye, it was possible to determine concentrations of the 10-fold, 100-fold and 1000-fold diluted samples. The concentration of the undiluted samples could not be established because the fluorescence corresponding to the highest point of the standard curve was lower than the fluorescence of undiluted samples. Measurements showed that the concentration of DNA in samples with different levels of fragmentation was influenced by the length of the fragments in all cases of sample dilutions. In case of 10-fold diluted samples, the DNA quantity in specimens with fragments of approximately 1500 bp was reduced only gently compared to intact samples (from 39.19 ± 7.79 to 37.07 ± 7.85), but concentrations of samples with targeted fragments of 500 bp and 150 bp were significantly reduced (from 39.19 ± 7.79 to 34.24 ± 7.62 or 27.73 ± 5.68 respectively). When it comes to 100-fold and 1000-fold dilutions of samples, significantly decreased concentrations were detected in all of the fragmented samples. Therefore, the amount of the DNA which was measured in samples decreased as the level of fragmentation increased. This decrease was statistically significant for 10-fold (p< 0.0001, F= 65.34), 100-fold (p< 0.0001, F= 152.6) as well as 1000-fold diluted samples (p< 0.0001, F= 109.8) (Figure 2).

**Figure 3:**
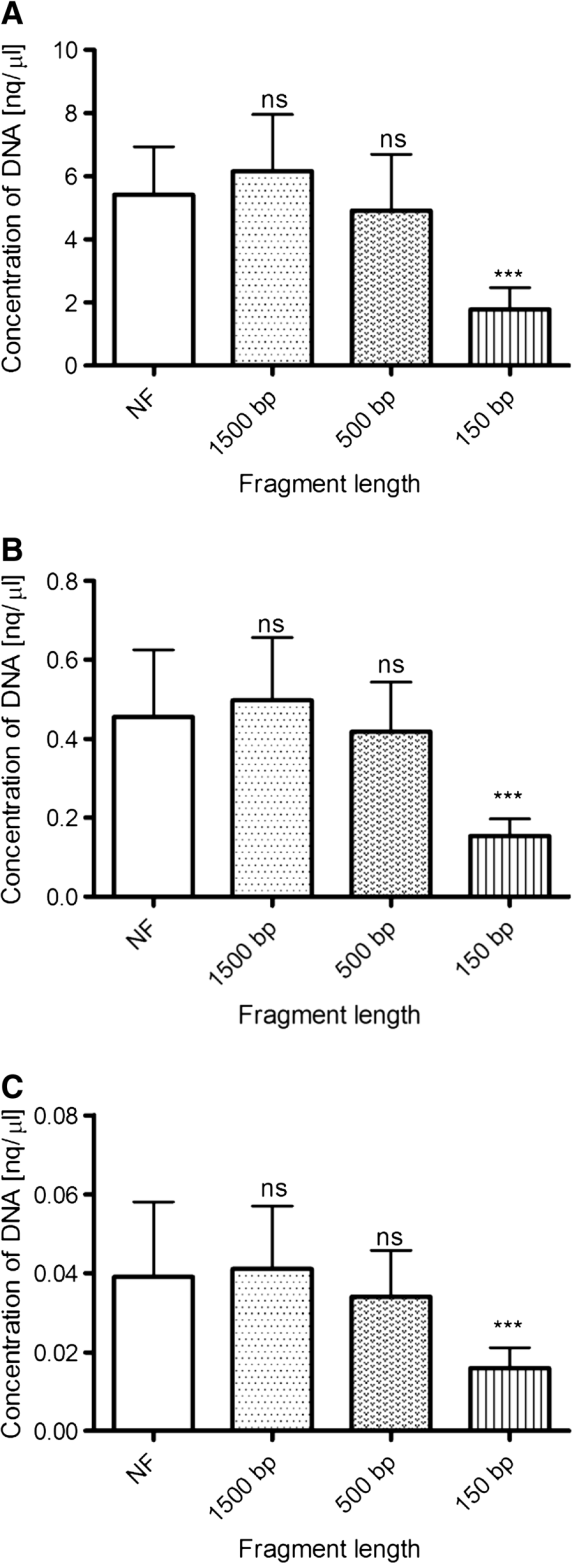
**The DNA quantification by qPCR with an Alu-based assay.** (**A**) The quantity of the most fragmented 10-fold diluted samples with a length of approx. 150 bp was significantly affected by the degree of the fragmentation. The same situation is shown in the case of 100-fold diluted (**B**) and 1000-fold diluted (**C**) samples as well. ns, statistically non-significant; ***, p< 0.0001.

Concerning the qPCR based DNA quantification, it was possible to determine the concentration of 10-fold, 100-fold and 1000-fold diluted samples, as for measurements with PicoGreen. Data from measurements of undiluted samples could not be evaluated because its concentration was above the highest point of the standard curve. Surprisingly, the quantity of DNA fragmented to approximately 1500 bp fragments slightly increased (p= ns) in comparison to unfragmented specimens. Conversely, samples with fragment lengths of approximately 500 bp showed lower (p= ns) concentrations than the control unfragmented specimens. However, the measured quantity of the most fragmented samples with lengths of approx. 150 bp was significantly affected by the degree of the fragmentation (p< 0.0001, F= 55.61, F= 27.05 and F= 10.74 for 10x, 100x and 1000x diluted samples, respectively) (Figure 3).

The concentration decrease for 10x diluted samples was from 5.42 ± 1.52 for unfragmented samples to 1.78 ± 0.69 for 150 bp fragmented samples (Figure 3A). All data regarding the DNA quantity measured by all methods are summarised in Table 1. For better illustration, the results are also displayed in the graphics shown in Figures 1, 2 and 3.

**Table 1:** Concentration of DNA measured by UV spectrophotometry, PicoGreen, and qPCR

	**NanoDrop**					
	**Undiluted samples**		**10-fold diluted samples**			
	**Mean ± SD**	**p value**	**Mean ± SD**	**p value**		
**intact DNA**	45.18 ± 9.99		7.77 ± 1.10			
**1500 bp**	41.39 ± 9.17	***	7.62 ± 1.30	ns		
**500 bp**	41.59 ± 8.76	***	7.26 ± 1.11	ns		
**150 bp**	44.41 ± 9.13	ns	7.70 ± 1.00	ns		
	**PicoGreen**					
	**10-fold diluted samples**		**100-fold diluted samples**		**1000-fold diluted samples**	
	**Mean ± SD**	**p value**	**Mean ± SD**	**p value**	**Mean ± SD**	**p value**
**intact DNA**	39.19 ± 7.79		6.19 ± 0.72		1.79 ± 0.20	
**1500 bp**	37.07 ± 7.85	ns	5.70 ± 0.72	***	1.62 ± 0.17	***
**500 bp**	34.24 ± 7.62	***	5.27 ± 0.68	***	1.46 ± 0.19	***
**150 bp**	27.73 ± 5.68	***	4.41 ± 0.44	***	1.20 ± 0.08	***
	**qPCR**					
	**10-fold diluted samples**		**100-fold diluted samples**		**1000-fold diluted samples**	
	**Mean ± SD**	**p value**	**Mean ± SD**	**p value**	**Mean ± SD**	**p value**
**intact DNA**	5.42 ± 1.52		0.46 ± 0.17		0.04 ± 0.02	
**1500 bp**	6.16 ± 1.79	ns	0.50 ± 0.16	ns	0.04 ± 0.01	ns
**500 bp**	4.92 ± 1.79	ns	0.42 ± 0.12	ns	0.03 ± 0.01	ns
**150 bp**	1.78 ± 0.69	***	0.15 ± 0.04	***	0.02 ± 0.01	***

The DNA concentrations are given in ng/μl. Results are presented as mean ± SD. The p-value represents the comparison to intact DNA sample. ns, statistically non-significant, ***, p< 0.0001.

## Discussion

Currently there are several methods that are commonly used to quantify DNA in solution; each has its pros and cons. The most commonly used methods are the quantification of DNA by measurement of the UV absorbance at 260 nm [5,9], fluorescence determination using fluorescent dyes [5,6,9,16] and qPCR-based assays [17,18]. The accuracy of measurement of the DNA concentration can be affected by several factors [16], one of those that is previously described is the length of DNA fragments [5,6,16,19]. Our study was focused on evaluation of the quantification of DNA using three different methods with respect to the level of DNA fragmentation.

First, the spectrophotometric measurement at 260 nm, which is the most frequently used method, was assessed. The Shokere et al. [5] group suggested that the concentration of DNA increased slightly with increasing fragmentation of DNA. However, our results show that DNA quantification based on A_260_ is not significantly affected by the level of DNA fragmentation (Figure 1). Nevertheless, the major disadvantages of this method are its low sensitivity and the fact that measurements do not discriminate between DNA and RNA and are biased by the presence of single-stranded DNA, oligonucleotides and free nucleotides as well [6,8]. On the other hand, A_260_ based quantification by NanoDrop is very simple and offers the fastest way of quantifying DNA, because no additional manipulation with the sample is necessary before measurement. Moreover, it enables the assessment of the amount of DNA and its purity in one step.

The second method addressed in our work was the measurement of fluorescence using the PicoGreen fluorescent dye. Previous studies indicated that DNA fragmentation significantly affects the quantification of DNA using PicoGreen, and measured DNA concentrations decrease with increasing DNA fragmentation [6,16]. Our results are in accordance with those studies, hence the accuracy of DNA quantification was also significantly affected by DNA fragmentation, as revealed by statistical analysis (Figure 2). Surprisingly, our measurements with PG assay overestimated the measured concentrations of samples by almost 10 times compared to other methods used in this study. This difference in measured concentration was caused by the use of Lambda DNA as a calibrator. Lambda DNA was used to create the calibration curve because it is supplied with the PG kit and recommended by the manufacturer. Therefore, it could be assume that most scientists measuring the concentration of DNA with PicoGreen use this DNA as a calibrator as well. When the human DNA with known concentrations was used as the control sample for the preparation of calibration curves on additional samples, the measured concentrations of unfragmented samples with all compared methods were concordant (data not shown).

As the third method, the quantitative real-time PCR with the Alu-based assay was used, which offers sensitivity for the DNA concentration measurements that are comparable to the PicoGreen assay or even higher. The extremely high sensitivity of this assay is based on the combination of the qPCR method and on abundance of the target sequence in human genome (>1x10^6^[20]). As in the case of PG, fragmentation affects the accuracy of concentration measurements significantly (Figure 3). The measurement deviation rose with the level of fragmentation in a fashion similar to PG. Smaller fragments resulted in lower concentrations measured. There was one exception to this observation, when the unfragmented specimens had lower concentrations measured in comparison to specimens which were fragmented to approx. 1500 bp. This exception was observed in all three dilutions and may have been the result of the complexity of unfragmented samples, when the effectiveness of amplification might be impaired by a worse accessibility of target sequences, e.g. because of secondary structures in complex DNA molecules. When unfragmented specimens were excluded from the statistical analysis of concentration measurements the statistically significant differences were confirmed between 1500 bp, 500 bp and 150 bp fragments (p<0.0001, F= 60.42, F= 40.18 and F= 15.98 for 10x, 100x and 1000x diluted samples, respectively). The differences regarding the decrease in measured concentration with respect to the fragmentation level could be the result of damage to the target sequences available for primer pair annealing and subsequent successful qPCR amplification. Therefore, measurement accuracy could also depend on the qPCR assay design, when shorter amplicons should be less affected. Nevertheless, the qPCR method showed more advantages than just an extremely high sensitivity. Other advantages include the identification of trace amounts of DNA (detection of less than 1 genomic equivalent is important in non-invasive prenatal diagnostics [21]), the detection of (q)PCR inhibitors in the analysed sample (important for forensic DNA analyses [22]) and the estimation of DNA fragmentation level when a combined Alu-based assay is implemented for DNA integrity detection [23] (which enables correction of fragmented DNA concentration measurement inaccuracy).

## Conclusions

Our work showed that the accuracy of concentration measurements based on fluorescent dyes and qPCR is influenced by the degree of DNA fragmentation, while this effect was not observed in DNA quantification by spectrophotometry. Therefore, we recommend quantifying intact DNA if possible. When quantifying fragmented DNA, an equally fragmented standard sample should be used to achieve the most reliable result.

## Methods

### Reagents

QIAamp DSP DNA Blood Mini Kit was obtained from Qiagen (Qiagen, Hilden, Germany). Quant-iT^™^ PicoGreen^®^ dsDNA Reagents and Kits was received from Invitrogen (Invitrogen – Molecular Probes, Eugene, OR, USA). AmpFLSTR^®^ Yfiler^™^ PCR Amplification Kit was obtained from Applied Biosystems (Applied Biosystems^™^ Warrington, UK). Primers were synthesised by Eurofins MWG Operon (Eurofins MWG Synthesis GmbH, Ebersberg, Germany). Maxima^®^ SYBR Green/ROX qPCR Master Mix (2x) was obtained from Fermentas (Fermentas, Vilnius, Lithuania).

### DNA isolation

Blood samples were gathered from 10 volunteers who also signed an informed consent. Blood samples were collected in K_3_EDTA blood collection tubes. Genomic DNA was isolated from 200 μl of blood using QIAamp DSP DNA Blood Mini Kit (Qiagen, Hilden, Germany) according to the manufacturer’s handbook following the blood and body fluid protocol. The DNA samples were dissolved in 150 μl of elution buffer. To obtain the needed concentration and necessary amount of DNA from all individuals, DNA isolation was performed in triplicate. As a result, the total starting volume of the processed blood samples was 600 μl and the total elution volume was 450 μl.

### DNA fragmentation

The DNA samples were fragmented by ultrasound with the use of Covaris S220 (Covaris, Woburn, MA, USA) in Snap-Cap microTUBEs with sample volume 130 μl. The water bath was cooled to 6°C during the fragmentation process. The settings used for targeted fragmentation were set-up according to the original manufacturer’s protocol and are listed in Table 2. Success of the fragmentation was assessed using agarose gel electrophoresis. After fragmentation, each of the 10 samples was available in four different lengths: unfragmented, fragmented with target peak at 1500 bp, fragmented with target peak at 500 bp and fragmented with target peak at 150 bp. Decimal dilutions of the samples (10-fold, 100-fold and 1000-fold diluted samples) were prepared and if possible used for subsequent measurements of DNA concentration.

**Table 2:** Summary of operating conditions for DNA shearing by ultrasound

	**Duty factor [%]**	**Peak incidence power [W]**	**Cycles per burst**	**Time [s]**
**1500 bp**	2	140	200	15
**500 bp**	5	105	200	80
**150 bp**	10	175	200	430

### DNA quantitation

The DNA concentration was quantified with three methods: absorbance measurement at 260 nm, fluorescence measurement with PicoGreen and qPCR with Alu-based assay.

Absorbance measurements at 260 nm were done using NanoDrop ND-1000 (Thermo Fisher Scientific Inc., Waltham, USA) with 1 μl of sample. This is the only method which also provided information about the purity of isolated DNA (using A_260/280_ ratio).

For DNA quantification with PicoGreen (PG), Quant-iT^™^ PicoGreen^®^ dsDNA Reagents and Kits (Invitrogen – Molecular Probes, Eugene, OR, USA) were used. An aqueous working solution of PG reagent was freshly prepared as a 1:200 dilution of the concentrated DMSO solution to 1x TE buffer (10 mM Tris–HCl, 1 mM EDTA, pH 7.5) prepared from 20x TE supplied by the manufacturer. 10 μl of DNA samples diluted in 90 μl of 1x TE buffer were mixed with 100 μl PG working solution to reach a final volume of 200 μl. The fluorometric measurements were performed using Tecan Safire 2 (Tecan, Grödig, Austria) at λ_ex_ 480 nm and λ_em_ 520 nm. Fluorescence of specimens was compared with fluorometric measurements from a standard sample. Lambda DNA supplied by the manufacturer served as standard sample at a stock concentration of 100 ng/μl. It was used to construct the standard curve by dilution to final concentrations of 2.5 ng/μl, 1 ng/μl, 500 pg/μl, 250 pg/μl, 100 pg/μl, 50 pg/μl, 25 pg/μl, 10 pg/μl and 5 pg/μl. The lowest possible concentration (fluorescence) of a standard sample that could be used was 25 pg/μl; therefore, points of the calibration curve under this concentration were excluded from the analysis.

To estimate DNA concentration of samples by quantitative real-time PCR (qPCR), an Alu-based assay was used according to the work of Umetani et al. [23]. The length of the analysed amplicon was 115 bp. All qPCR reactions were performed on the Eppendorf realplex^4^ Mastercycler ep gradient S (Eppendorf, Hamburg, Germany) and analysed using the Eppendorf Realplex 2.0 software. PCR reactions were prepared containing 1x Maxima^®^ SYBR Green/ROX qPCR Master Mix (Fermentas, Vilnius, Lithuania), 0.3 μM primers, 1 μl of DNA and nuclease-free water to reach final volume 15 μl. The PCR program was as follows; initial denaturation step at 95°C for 5 minutes, then the reaction was cycled for 40 cycles, each containing denaturation at 95°C for 15 seconds and combined annealing/extension step at 60°C for 1 minute. The PCR reaction was finished by melting curve analysis consisting of a denaturation at 95°C for 15 seconds, melting start at 60°C for 15 seconds followed by a continual increase of temperature up to 95°C for 20 minutes and final denaturation at 95°C for 15 seconds. The standard curve for qPCR assay was generated from human Control DNA 9947A from AmpFLSTR^®^ Yfiler^™^ PCR Amplification Kit (Applied Biosystems^™^, Warrington, UK) with stock concentration 10 ng/μl that was diluted to 1 ng/μl, 0.1 ng/μl and 0.01 ng/μl in nuclease-free water. All measurements with the three methods were performed in duplicate.

### Statistical analysis

Differences between groups were tested using one-way analysis of variance (ANOVA) for repeated measures test with post-test Tukey modified *t*-test. Data analysis was performed using GraphPad Prism 5.0 software and Microsoft Excel 2007^®^. P<0.05 was considered statistically significant. Data are presented as mean ± SD.

## Abbreviations

cNA: circulating Nucleic Acid; qPCR: quantitative real-time PCR; EDTA: Ethylenediaminetetraacetic Acid; DMSO: Dimethyl Sulphoxide.

## Competing interests

The authors declare that they have no competing interests.

## Authors’ contributions

TS collected the samples, performed DNA fragmentation and DNA quantitation, and drafted the manuscript. GR was involved in sample collection, performed DNA isolation and contributed to DNA quantitation. PC conceived the study design and performed the statistical analysis. TS summarised the results and performed manuscript critical review. GM prepared protocols and methods of the study, performed manuscript critical review and supervision of the study, and revised the draft manuscript. All authors read and approved the final manuscript.
